# Humoral Immunity Profiling of Subjects with Myalgic Encephalomyelitis Using a Random Peptide Microarray Differentiates Cases from Controls with High Specificity and Sensitivity

**DOI:** 10.1007/s12035-016-0334-0

**Published:** 2016-12-15

**Authors:** Sahajpreet Singh, Phillip Stafford, Karen A. Schlauch, Richard R. Tillett, Martin Gollery, Stephen Albert Johnston, Svetlana F. Khaiboullina, Kenny L. De Meirleir, Shanti Rawat, Tatjana Mijatovic, Krishnamurthy Subramanian, András Palotás, Vincent C. Lombardi

**Affiliations:** 1 Nevada Center for Biomedical Research, 1664 N Virginia St. MS 0552, Reno, NV 89557-0552 USA; 2 The Biodesign Institute Center for Innovations in Medicine at Arizona State University, Tempe, AZ USA; 3 Department of Biochemistry and Molecular Biology, University of Nevada, Reno, NV USA; 4 Nevada INBRE Bioinformatics Core, University of Nevada, Reno, NV USA; 5 Tahoe Bioinformatics, Incline Village, Reno, NV USA; 6 Kazan Federal University, Kazan, Russian Federation; 7 R.E.D. Laboratories, Zellik, Belgium; 8 Asklepios-Med (private medical practice and research center), Kossuth Lajos sgt. 23, Szeged, 6722 Hungary; 9 Department of Pharmacology, University of Nevada, Reno, School of Medicine, Reno, NV USA

**Keywords:** Antibody, Chronic fatigue syndrome, Immunosignature, Myalgic encephalomyelitis, Peptide array

## Abstract

Myalgic encephalomyelitis (ME) is a complex, heterogeneous illness of unknown etiology. The search for biomarkers that can delineate cases from controls is one of the most active areas of ME research; however, little progress has been made in achieving this goal. In contrast to identifying biomarkers that are directly involved in the pathological process, an immunosignature identifies antibodies raised to proteins expressed during, and potentially involved in, the pathological process. Although these proteins might be unknown, it is possible to detect antibodies that react to these proteins using random peptide arrays. In the present study, we probe a custom 125,000 random 12-mer peptide microarray with sera from 21 ME cases and 21 controls from the USA and Europe and used these data to develop a diagnostic signature. We further used these peptide sequences to potentially uncover the naturally occurring candidate antigens to which these antibodies may specifically react with in vivo. Our analysis revealed a subset of 25 peptides that distinguished cases and controls with high specificity and sensitivity. Additionally, Basic Local Alignment Search Tool (BLAST) searches suggest that these peptides primarily represent human self-antigens and endogenous retroviral sequences and, to a minor extent, viral and bacterial pathogens.

## Introduction

Myalgic encephalomyelitis (ME), also commonly referred to as chronic fatigue syndrome or ME/CFS, is a heterogeneous illness characterized by a number of physical symptoms and comorbid conditions including neurocognitive dysfunction, systemic inflammation, innate immune activation, and gastrointestinal abnormalities [1]. Current estimates suggest that as many as 2.5 million individuals suffer from ME, with an annual productivity loss in excess of US$9 billion in the USA alone, underscoring the importance of ME as a major public health concern both economically and socially.

Exactly what causes ME is unknown at this time; however, a number of potential triggers are associated with the development of the disease including physical trauma, emotional distress, infection, and chemical exposure [2–4]. Familial studies and genetic screening studies indicate that a genetic predisposition also plays an important role in the pathophysiology of ME [5–8]. Presently, there are no unique physical symptoms or reproducible biomarkers that can delineate this disease. For this reason, a diagnosis can only be made when an individual meets a series of inclusion and exclusion criteria, typically through a lengthy and expensive diagnostic process [9, 10]. Although the search for potential biomarkers has been one of the most active areas of ME research, little progress has been made in achieving this goal. Here, we report the progress in applying the immunosignature technology to this problem.

While not universally prevalent, a number of clinical observations such as natural killer (NK) cell dysfunction, viral reactivation, and inflammatory cytokine production have been consistently reported in the ME literature over the years and support an organic basis for this disease [11–15]. However, the mechanisms responsible for these observations remain elusive, but, if identified, this knowledge would lead to a greater understanding of ME pathology, potentially leading to effective treatment options.

Antibodies are glycoproteins, produced by B lymphocytes (B-cells) and plasma cells, in response to foreign molecules (antigens), such as those found in bacteria and viruses. As the central component of humoral immunity, they limit the spread of infection by binding to and neutralizing the pathogen or by activating other adaptive immune responses. B-cells also produce antibodies directed against self-antigens, but they are normally removed in the bone marrow early in their development. Although on rare occasion, however, this system fails, leading to autoimmunity.

Identifying the antigens to which antibodies react with during the course of a disease may lead to a greater understanding of the humoral immune response associated with that disease. For instance, ascertaining a dominant epitope may help in the development of an effective vaccine or identifying reactive antigens that are homologous to self-proteins may reveal autoimmune pathology [16]. Immunosignatures (IMS) are screens of serum antibodies using random peptide arrays, and this technique has been used successfully to identify biomarkers in diseases that are difficult to diagnose such as cancer, valley fever, and Alzheimer’s disease [17–19].

In the present study, we utilized a microarray consisting of 125,000 random peptide sequences to screen the serum of healthy control subjects and those who present with symptoms consistent with a diagnosis of ME. Our data identified an IMS that accurately delineated ME cases from controls with 92.9% specificity and 97.6% sensitivity. Additionally, Basic Local Alignment Search Tool (BLAST) searches suggest that these peptides have sequence homology primarily to human self-antigens and endogenous retroviral sequences, but also to a minor extent, viral and bacterial antigens. This proof-of-concept study potentially represents the first step toward a specific and sensitive diagnostic for ME and also may provide important knowledge regarding the pathophysiology of the disease.

## Materials and Methods

### Study Subjects

For this study, a total of 42 subjects were recruited from across the USA and Europe. ME cases consisted of 11 US and 10 European subjects and controls consisted of 12 US and 10 European subjects. Informed consent was obtained from each participant according to a human subjects protocol approved by the University of Nevada Biomedical Institutional Review Board (protocol B12-031). The cases identified as having ME were physician diagnosed and met Carruthers et al.’s criteria for ME as well as 1994 Fukuda et al.’s criteria for CFS [9, 10, 20].

### Microarray

Serum samples from respective cases and controls were diluted 1:1 in glycerol and stored at −20 °C until analyzed. The 125,000 random 12-mer peptide microarrays were manufactured according to the methods of Leguti et al. [21] and blocked in 0.5% BSA (Sigma, St. Louis, MO) and 1× PBS, pH 7.2. Samples were diluted to 1:1000 in 1× PBS, 0.5% BSA, and 0.05% Tween 20 pH 7.2 and exposed to the microarrays for 1 h at 37 °C with gentle agitation. After 1 h, the arrays were washed in distilled water 3× and incubated with 4 nM of Alexa Fluor 555 conjugated goat anti-human IgG (H & L) and 5 nM of Alexa Fluor 647 conjugated goat anti-human IgM heavy chain (Thermo Fisher) for 1 h at room temperature, then washed 3× in distilled water and 1× in 90% isopropyl alcohol, and dried in a centrifuge. Slides were scanned on an Innopsys 1100 scanner at 0.5-um resolution, and TIFF images were aligned in GenePix 6.0.

### Data Analysis

Peptide expression data were subjected to the following quality control steps. First, array images were evaluated for clearly identifiable spatial variation, including streaks and bubbles. Peptide array background values were subtracted from signal values in both Cy3 and Cy5 channels using simple background subtraction. Before normalization, peptides with an incidence of high background values were filtered. Specifically, peptides having more than 50% incidence in either channel of negative background-corrected values (signal background) were excluded. The remaining raw values were normalized first within each array via the median method and then between arrays using the Aquantile method, with the *limma* package in R [22].

Normalized data were then averaged across replicated peptides and replicated samples. Peptides were again filtered after normalization and averaging for high incidence of low signal intensities with respect to background intensities. (These are seen as missing values in the data, as normalization includes a logarithmic transform that is not applicable to negative values.) Specifically, any peptide having more than 25% missing values for either cohort was excluded.

This final data set (103,385 peptides) was analyzed using the data mining algorithm Random Forest [23] in a progressive stepwise process of reduction using each respective peptide sequence as the predictive variable and subject status (ME case or control) as the target variable. For each iteration, 5000 random decision trees were built using one half the square root of *N* with a minimal of two parental nodes at each branch. Small classes were upweighted to equal the size of the largest target class and “out of bag” testing with replacement was employed to test the model. In the first step, the top 30% of peptides were selected and rescreened; then, the top 40% of peptides were rescreened. In the final step, multiple iterations were preformed systematically, removing the least contributing peptides until the signature did not improve.

In order to potentially identify the biological antigens to which the synthetic random peptides represent, the penultimate iteration, consisting of 233 peptides, was searched against viral, bacterial, human, and endogenous retroviral proteins, each derived from the National Center for Biotechnology Information (NCBI) nr database using the ncbi-blast+ BLASTP protein sequence similarity search tool (v. 2.4.0). The virus protein database was produced by filtering nr for virus species with human hosts as recorded at NCBI Taxonomy. Similarly, the bacterial protein database was generated by restriction of nr to the subset of bacterial species identified within the PATRIC database to be associated with human hosts (http://www.patricdb.org). The human protein database contained those found in NCBI RefSeq. The HERVd protein database was generated by the combination of nr proteins self-identified in human endogenous retroviral lineages with a set of human endogenous retrovirus (HERV)-like proteins reported as proteins of *Homo sapiens* origin. BLAST parameters were set as follows: wordsize 2, window_size 15, threshold 16, PAM30 scoring matrix, gapopen 9, gapextend 1, evalue 1000, maximum reported alignments per high scoring pair (HSP) of query/subject (max_hsps) 1, and minimum query coverage by HSP percent (qcov) 34. Additional BLAST output format options were set to record NCBI taxonomic identifiers (taxids) of proteins and the BLAST traceback operations (btop), a text string that encodes the alignment, mismatch, and gap information. Hits lacking any ungapped subalignment of five or more amino acid identities were identified using btop information and excluded from the analysis set. Species and genus taxa of subject proteins were mapped to each protein from the reported taxids with ETE Toolkit (http://etetoolkit.org; v3.0.0b35); a Python framework for phylogenetic tree analysis. In order to limit biasing as a result of protein size, we implemented a simple metric adjustment (Adj.), whereby the number of amino acids in a given protein was divided by the number of peptides having homology to that protein. Potentially conserved peptide motifs were investigated using the multiple sequence alignment tool Clustal X [24].

## Results

### Classification by Random Forest

In order to test whether differences exist between the antibody profiles of ME cases and controls, analysis was carried out using the Random Forest (RF) classification algorithm. The RF algorithm uses an ensemble of unpruned classification or regression trees produced through bootstrap sampling of the training data set and random feature selection in tree generation. Prediction is made by a majority vote of the predictions of the ensemble. The strength of the analysis was evaluated by out of bag sampling with replacement of the original data. RF is an attractive method since it handles both discrete and continuous data, it accommodates and compensates for missing data, and it is invariant to monotonic transformations of the input variables. The RF algorithm is well suited for peptide microarray analysis in that it can handle highly skewed values well and weighs the contribution of a given peptide according to its relatedness with others.

**Fig. 1:**
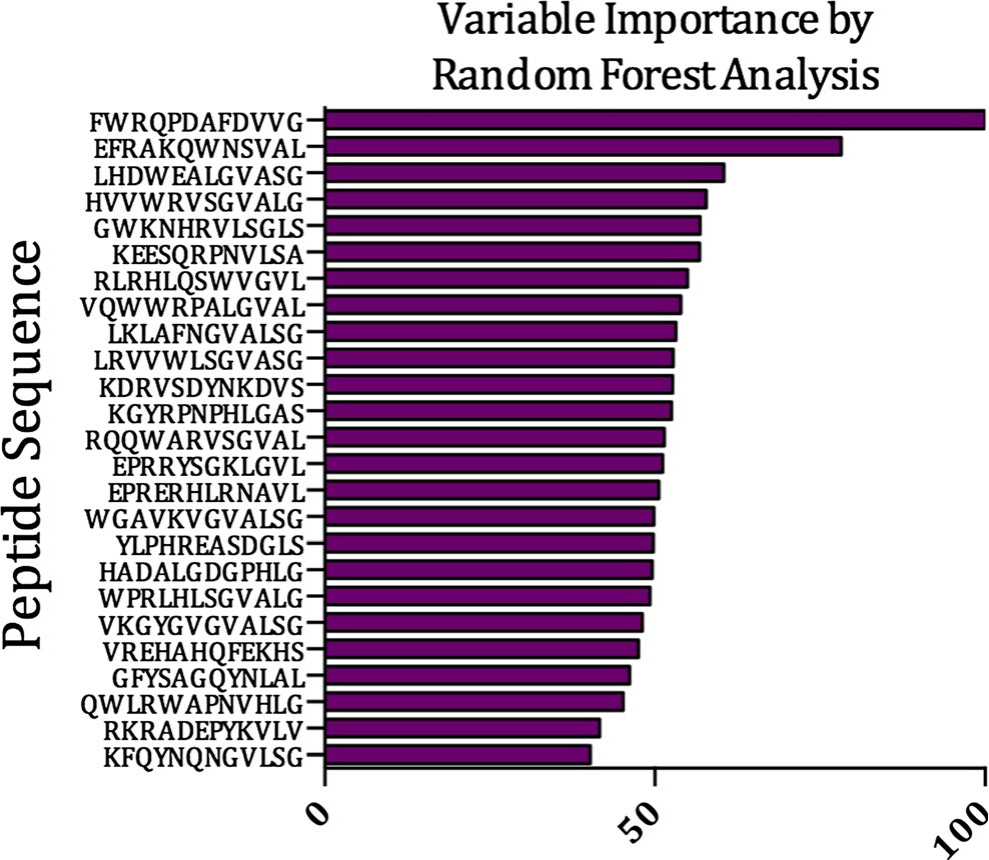
Random forest prediction. *Horizontal bars* represent the relative importance that each random peptide contributes to the final diagnostic signature

Through multiple iterations of RF processing, we identified a signature of 25 peptides that was able to identify ME cases from controls with 92.9% specificity and 97.6% sensitivity (Table 1). Each peptide was ranked according to its contribution to the signature, with the top peptide being ranked at 100 and subsequent peptides ranked relative to this peptide. The relative contribution of these 25 peptides and their sequence are given in Fig. 1. We conclude that, at least based on the analysis with this small sample set, IMS can distinguish ME from non-ME samples.

**Table 1:** Results of 21 ME cases and 21 controls each screened for reactivity with IgG and IgM

Actual class	Total class	Percent class	Predicted classes	
			Control *N* = 44	ME case *N* = 44
Control	42	97.62%	41	1
ME case	42	92.86%	3	39
Total	84			
Average		95.24%		
Overall % correct		95.24%		
Specificity		92.86%		
Sensitivity/recall		97.62%		
Precision		93.18%		
F1 statistic		95.35%

### Homology BLAST Search and Sequence Alignment

In order to potentially identify the biological antigens to which the synthetic random peptides may represent, we developed an analytical pipeline and used this to search the 233 peptides from our penultimate RF iteration against the human proteome. Additionally, we have previously reported that gastrointestinal plasmacytoid dendritic cells (pDCs) produce proteins that are consistent with human endogenous retroviral sequences [25]; therefore, we also included these sequences in our search. Finally, we used this pipeline to search for homology to bacterial and viral antigens of pathogens known to infect humans.

Our initial analysis identified over 5000 human protein sequences that met our search criteria. When filtered to limit those proteins that were identified by four or more random peptides, this number was reduced to 166 proteins. In an attempt prevent overrepresentation of larger proteins, which have a greater chance of having homologous sequences to a given random peptide, we used the simple metric of dividing the number of amino acids of the protein by the number of peptides that were homologous to that protein. The top 30 human proteins, adjusted for size, are given in Table 2. Among the likely most relevant human proteins identified in this search were proteins involved in mitochondrial function (AMACR, ETFDH, SLC25A40), lipid metabolism (AGK, ACOXL, CEL SEC23A), neurological function (APBA3, ASIC1, GABRB3, STAC), and immune responses (CD274, LGMN, MX1, MX2).

**Table 2:** Number of peptides homologous to a respective human protein sequences

Peptides	Accession	Symbol	Description	Length	Adj.
4	Q8NAP1	GATS	Stromal antigen 3 opposite strand	163	40.8
13	NP_033173	SEC23A	Sec23 homolog A	765	58.8
10	AKI70819	ETFDH	Electron transfer flavoprotein Dehydrogenase	617	61.7
9	NP_073150	EBF2	Early B-cell factor 2	575	63.9
5	AAH27322	SLC25A40	Solute carrier family 25 Member 40	338	67.6
5	EAX02872	ARMCX4	Armadillo repeat containing, X-linked 4	360	72
4	EAW58763	CD274	CD274 molecule	290	72.5
7	NP_056530	PLA2G3	Phospholipase A2 group III	509	72.7
7	Q6PJ69	TRIM65	Tripartite motif containing 65	517	73.9
4	NP_001103408	C6orf136	Chromosome 6 open reading frame 136	315	78.75
4	CAG46638	HMOX2	Heme oxygenase 2	316	79
7	NP_004877	APBA3	Amyloid beta precursor Protein binding family A member 3	575	82.1
9	P19835	CEL	Carboxyl ester lipase	753	83.7
7	NP_001171517	MX1	MX dynamin-like GTPase 1	662	94.6
4	ABQ59031	AMACR	Alpha-methylacyl-CoA racemase	382	95.5
4	NP_003140	STAC	SH3 and cysteine-rich domain	402	100.5
6	NP_001307526	DDX5	DEAD-box helicase 5	614	102.3
4	NP_060708	AGK	Acylglycerol kinase	422	105.5
5	NP_001086	ASIC1	Acid sensing ion Channel subunit 1	528	105.6
7	NP_758441	CTAGE1	Cutaneous T-cell Lymphoma-associated antigen 1	745	106.4
5	CAG33352	CCT2	Chaperonin containing TCP1 subunit 2	535	107
4	CAG33687	LGMN	Legumain	433	108.3
10	NP_006217	PLCL1	Phospholipase C-like 1	1095	109.5
5	NP_000449	HNF1B	HNF1 homeobox B	557	111.4
4	NP_068712	GABRB3	Gamma-aminobutyric acid type A receptor Beta3 subunit	473	118.25
6	P20592	MX2	MX dynamin-like GTPase 2	715	119.7
7	NP_060868	CACNA2D3	Calcium voltage-gated channel auxiliary subunit alpha2delta 3	1091	121.2
5	P30825	SLC7A1	Solute carrier family 7 member 1	629	125.8
4	NP_004557	PFKFB3	6-Phosphofructo-2-kinase/fructose-2,6-biphosphatase 3	520	130
4	XP_011509718	ACOXL	Acyl-CoA oxidase-like	547	136.8

**Fig. 2:**
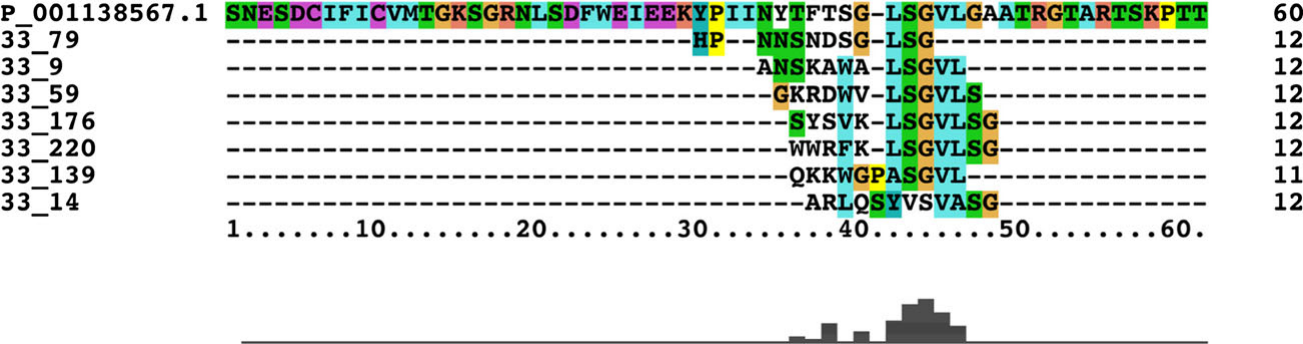
Clustal X alignment of seven random peptides homologous to HERV-H LTR-associating protein 1 precursor

**Fig. 3:**
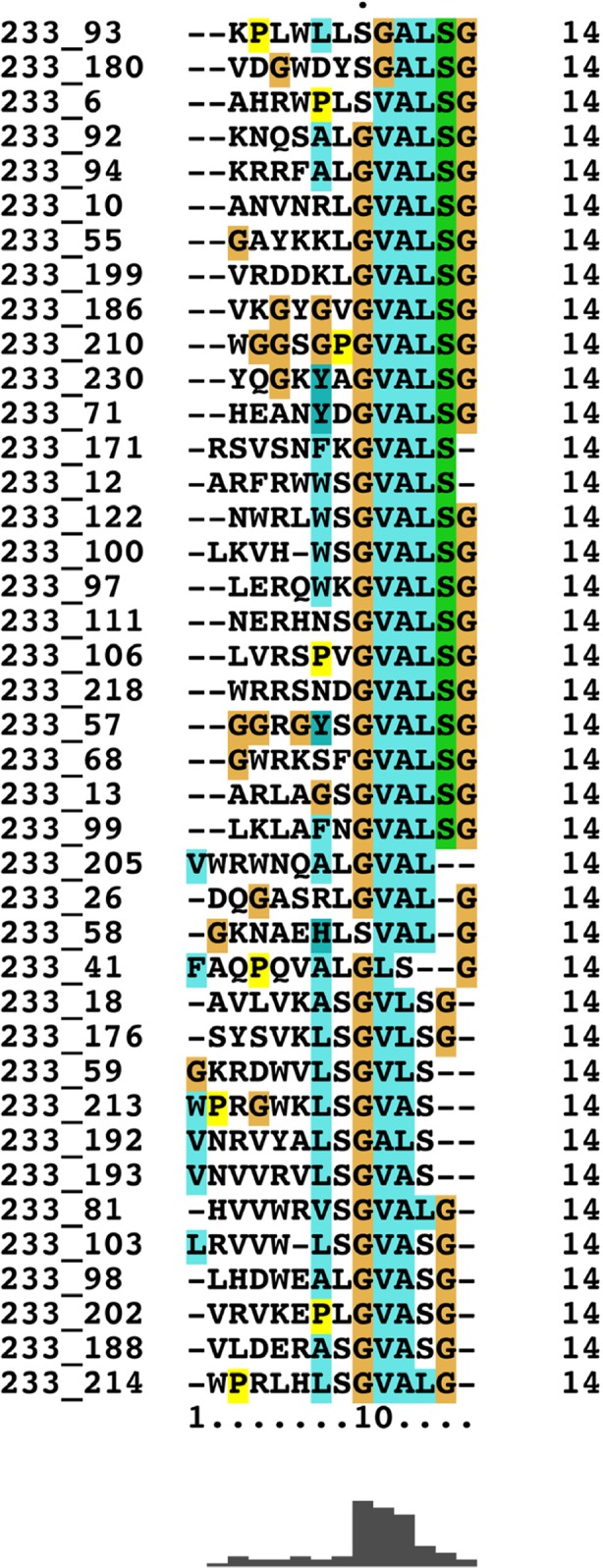
Clustal X alignment. Clustal X alignment of 40 random peptides showing the largely conserved motif of GVALSG

Previous studies have proposed that HERV elements may be associated with neurological diseases including multiple sclerosis [26], amyotrophic lateral sclerosis [27], and schizophrenia [28]. With this, and our previous studies in mind, we included HERV sequences in our homology search. Nine HERV sequences were identified with sequence homology to at least two of our top 233 random peptide sequences; the most relevant HERV sequence showed homology to seven of the 233 peptides (Table 3). Importantly, the seven sequences were not randomly represented throughout the HERV sequence but largely converged the same position in the protein, as revealed by Clustal X alignment (Fig. 2). Further analysis showed that this conserved motif is well represented in 40 of the 233 random peptides (Fig. 3), suggesting that this motif significantly contributes to the observed IMS.

**Table 3:** Number of peptides homologous to respective human endogenous retroviral sequences

Peptides	Accession	Symbol	Description	Length	Adj.
7	NP_001138567	HHLA1	HERV-H LTR-associating protein 1 precursor	531	75.8
3	P61566	ERVK-24	Endogenous retrovirus group K member 24	588	196
3	AAY87455	ERVK-6	Env type 1, partial	603	201
4	AAM81188	HRV-5	Pol protein, partial	863	215.7
3	P61570	ERVK-25	Endogenous retrovirus group K member 25	661	220.3
3	P61565	ERVK-21	Endogenous retrovirus group K member 21	698	232.6
2	P60507	ERVFC1	Endogenous retrovirus group FC1 env polyprotein	584	292
2	Q14264	ERV3–1	Endogenous retrovirus Group 3 member 1	604	302
2	ABB52637	ERVPABLB-1	Endogenous retrovirus group PABLB member 1	665	332.5

Immunoreactivity to a given synthetic random peptide may be the result of cross-reactivity to pathogen-derived antigens encountered during an infection. To explore this possibility, we surveyed our top 233 random peptides against the proteomes of bacteria and viruses known to infect humans (Table 4). As before, the proteins were filtered to limit those that were hit by multiple peptides; however, the threshold was reduced to three peptides. When adjusted for protein size, the six most significant viral proteins with sequence homology to our random peptides were the gp120 protein of HIV (six hits); followed by the polyprotein of GB virus Ccpz (three hits); the envelope glycoprotein I of Human herpesvirus 2 (four hits); the phosphoprotein of canine distemper virus (four hits); the RNA-dependent RNA polymerase, rodent paramyxovirus (three hits); and finally, the outer capsid protein of porcine rotavirus C (three hits). When adjusted for protein size, the most significant bacterial peptides with sequence homology to our top random peptides were a hypothetical protein from *Serratia marcescens* (four hits), a diaminopimelate aminotransferase from *Paenibacillus senegalensis* (five hits), the peptidase M16 of *Anaerofustis stercorihominis* (three hits), the type IV secretion protein Rhs of *Hafnia alvei* (three hits), and the SusC/RagA family TonB-linked outer membrane protein of *Bacteroides nordii* (three hits). Numerous other human pathogens were identified that contained homologous sequences to our random peptides but were excluded because of our adjusting metric (data not shown).

**Table 4:** Number of peptides homologous to a respective viral and bacterial protein sequences

Peptides	Accession	Symbol	Description	Length	Adj.
Viruses					
6	CAD87195	Q70QU7	gp120 protein, human immunodeficiency virus 1	198	33
3	ADL29714	E0ADF8	Polyprotein GB virus Ccpz	242	80.7
4	AMB66322	P06764	Envelope glycoprotein I human herpesvirus 2	372	93
4	AJO72800	P06940	Phosphoprotein canine distemper virus	507	126.8
3	BAO04373	U6C7N8	RNA-dependent RNA polymerase rodent Paramyxovirus	549	183
3	AJL35076	A0A0C5B2I7	Outer capsid protein Porcine rotavirus C	733	244.3
Bacteria					
4	WP_033638106	None	Hypothetical protein [*Serratia marcescens*]	284	71
5	WP_010273745	None	diaminopimelate aminotransferase [*Paenibacillus senegalensis*]	397	79.4
3	WP_007050391	B1C8L1	Peptidase M16 [*Anaerofustis stercorihominis*]	422	140.7
3	WP_004091469	G9Y4S5	Type IV secretion protein Rhs [*Hafnia alvei*]	644	214.7
3	WP_007483701	I9H2D3	SusC/RagA family TonB-linked outer membrane protein [*Bacteroides nordii*]	1046	348.7

## Discussion

In this report, we present a “proof-of-concept” study where random peptide arrays show utility in delineating ME cases from healthy controls. The ultimate goal of this work is the development of a non-subjective clinical tool for diagnosing patients with ME. To this end, we utilized a random peptide array, which has previously produced IMS for other chronic and complicated diseases that are difficult to diagnose such as cancer, valley fever, and Alzheimer’s disease [17–19]. Given the complexity and the size of the data set, we elected to use the machine learning data mining algorithm Random Forest to identify potential candidates that may lead to a diagnostic signature. Using out of bag testing with replacement, our model was able to predict cases and controls with 92.9% specificity and 97.6% sensitivity using 25 peptides. Finally, we developed an analytical pipeline to BLAST these peptides against the human, HERV, virus, and bacterial proteomes for sequence homology, in order to explain the underpinnings of the IMS. This pilot study supports the premise that immunosignatures represent a viable approach to achieve our overarching goal of developing a diagnostic tool and further potentially identify naturally occurring antigens to these antibodies.

A number of studies have attempted to identify a reproducible biomarker for ME [29–33]. In particular, serum or plasma cytokine and chemokine analyses have shown promise, in that several investigators, including our group, have reported clear differences when comparing ME cases to healthy controls [11, 15, 34–36]. Additionally, cytokine differences may provide valuable information regarding the pathophysiology of the disease. For instance, previous studies have suggested that ME is characterized by a Th2 shift [37, 38], an observation that may explain the prevalence of persistent viral infections associated with this disease [39]. However, most cytokines that are produced in response to innate immune activation, as is seen with ME, are not consistently expressed [40]. In contrast, serum antibodies are much more stable. For example, most IgG subclasses have half-lives of more than 20 days [41].

Immunosignatures have been used successfully to provide understanding to the pathophysiology of chronic diseases. For example, Restrepo et al. reported that plasma antibodies from subjects with Alzheimer’s disease (AD) could be used to provide an IMS that can distinguish AD cases from non-AD controls reproducibly over time [42]. It was also shown that eight of the 50 signature random peptides have the ability to react with antibodies initially raised against native amyloid-β [19], a protein shown to be significantly involved in Alzheimer’s disease [43].

These observations raise the possibility that an IMS may provide clues to the pathophysiology of ME. However, in contrast to AD, there are no proteins known to be ubiquitous in the pathological process of ME with which to test. Although we have identified a number of peptides that accurately identify ME cases from controls, divining naturally occurring homologous peptides is challenging. An antibody typically covers approximately 15 amino acids of its cognate epitope; however, only about five or six amino acids contribute to the ΔG° of antigen/antibody binding [44, 45] and these amino acids may not necessarily be contiguous. Therefore, the natural antigen is likely to be very short and may contain gaps in the primary sequence. In an attempt to overcome these obstacles, we developed a custom analytical pipeline to conduct BLAST searches against the NCBI human protein database. In addition to annotated human proteins, we also queried against HERV proteins as well as proteins from bacteria and viruses known to infect humans. As it was probable that very large proteins would be more likely to be identified over smaller proteins by random chance, we implemented a metric to adjust for this issue. Interestingly and somewhat unexpectedly, we identified a number of protein hits in the human database. Previous studies by Fluge et al. showed that anti-CD20 B-cell depleting drug, rituximab, showed efficacy in treating subjects with ME [46, 47]. Although the mechanism responsible for this observation remains to be elucidated, it does suggest that self-reactive antibodies may contribute to the pathophysiology of this disease. Indeed, several studies have reported self-reactive antibodies in subjects with ME [48–50] so our results are potentially consistent with this supposition.

Previously, we reported that gut-associated pDCs in subjects with ME were immunoreactive to antibodies that react with endogenous retroviral proteins [25]. Other studies have also reported retroviral protein sequences in subjects with neurological and autoimmune disease; however, the meaning of these observations has yet to be resolved [28, 51, 52]. Nonetheless, these sequences, if uniquely expressed in a disease state, may prove to be a useful biomarker. We thus included the HERVd databases in our query space and observed seven of our significant peptide-1displayed sequence homology to the HERV-H LTR-associating protein 1 precursor (HHLA1). If dispersed through the protein, the probability of seven random peptides hitting this sequence would be exceedingly small. However, upon further examination, it was discovered that all seven peptides represented a largely conserved sequence (LSGVLS) in the HERV protein. A similar and overlapping conserved sequence motif (GVALSG) was observed in at least 40 of the 233 top peptides identified by our RF analysis. This observation raises two important issues. First, the discovery of this conserved peptide motif may represent a critical discovery in resolving the pathophysiology of ME, assuming it is confirmed in other cohorts and it is shown to be unique to ME. Secondly, because this motif is short and present in many pathogens, we cannot say with absolute certainty that we have identified the naturally occurring antigen that gave rise to the antibodies that react with this motif. When this sequence is considered in isolation, we have observed it within several other proteins, in particular, the bacteria genus *Burkholderia* and also in the human protein calcium voltage-gated channel protein CACNA2D3 (Table 2). Further studies will be required to identify with greater certainty the native antigen to this conserved motif.

Lastly, we BLASTed the 233 random peptides identified by RF against the proteomes of viruses and bacteria known to infect humans. The most prevalent viral hit was to the gp120 protein of human immunodeficiency virus 1. This sequence was homologous to the conserved motif; therefore, it is likely the result of cross-reactivity to antibodies raised to HERVs or another similar sequence. Of the bacterial hits that have been previously associated with ME, the type IV secretion protein Rhs of *H. alvei* was identified in our search. *H. alvei* is Gram-negative, facultative anaerobic intestinal bacteria. A previous study by Maes et al. reported that ME cases have elevated serum IgA and IgM antibodies, to *H. alvei*, that likely result from intestinal bacterial translocation [53]. Given the diversity of gastrointestinal bacteria, it may be possible that antibodies raised to translocated bacterial products could potentially cross-react with self-proteins. Indeed, a number of studies have identified gastrointestinal comorbidity and/or an altered gut microbiome as a potential associating factor with ME [54–58]. However, at this point, it has yet to be determined if these observations are cause or effect.

In conclusion, the data presented in this report represents a proof-of-concept study that random peptide arrays show utility in delineating ME cases from healthy controls. Additionally, our study has identified a conserved peptide motif that is preferentially recognized by serum antibodies in a large number of ME cases over that of healthy controls. This study warrants further investigations using additional ME cohorts as well as cohorts of subjects with other chronic diseases with overlapping symptomology.
